# The Relationship of Metabolic Syndrome Traits with Beta-Cell Function and Insulin Sensitivity by Oral Minimal Model Assessment in South Asian and European Families Residing in the Netherlands

**DOI:** 10.1155/2016/9286303

**Published:** 2016-08-11

**Authors:** Thekla Geragotou, Sjaam Jainandunsing, Behiye Özcan, Felix W. M. de Rooij, Alexander Kokkinos, Nicholas Tentolouris, Eric J. G. Sijbrands

**Affiliations:** ^1^Department of Internal Medicine, Erasmus MC-University Medical Center Rotterdam, 3015 CE Rotterdam, Netherlands; ^2^First Department of Propaedeutic and Internal Medicine, Medical School, National and Kapodistrian of Athens, Laiko General Hospital, 11527 Athens, Greece

## Abstract

*Background*. There are different metabolic syndrome traits among patients with different ethnicities.* Methods*. We investigated this by studying 44 South Asians and 54 Europeans and classified them in three groups according to the occurrence of metabolic syndrome (MetS) and Type 2 Diabetes (T2D). Insulin sensitivity index (ISI), static, dynamic, and total beta-cell responsivity indices (Φ), and disposition indices (DIs) were calculated with the use of oral minimal model (OMM).* Results*. In both ethnicities, ISI was lower in the subgroup with MetS and T2D as compared to the subgroup without MetS nor T2D (*P* < 0.004). South Asians without MetS were more insulin resistant than Europeans without MetS (*P* = 0.033). In the South Asians, ISI, dynamic DI, and static DI were associated significantly (*P* < 0.006) with high-density lipoprotein cholesterol and triglycerides. In the Europeans, ISI was associated with waist-to-hip ratio (*P* = 0.005) and systolic and diastolic blood pressure (*P* < 0.005), while static DI was related to the systolic blood pressure (*P* = 0.005).* Conclusions*. MetS was linked with insulin resistance and reduced capacity to handle glucose regardless of ethnicity. ISI and DIs were associated with lipid traits in South Asians and with blood pressure in Europeans suggesting that insulin resistance enhances different metabolic syndrome traits among different ethnicities.

## 1. Introduction

Overweight and physical inactivity enhance each other and decline the sensitivity to insulin. Resistance to insulin is characteristic of metabolic syndrome (MetS), which is defined as a cluster of the following cardiovascular risk factors: central obesity, impaired glucose tolerance, dyslipidemia, and hypertension. MetS constitutes a major health problem, as it is strongly associated with type 2 diabetes mellitus (T2D) and cardiovascular disease [1–3]. Furthermore, insulin resistance is a consistent finding in T2D and appears to contribute to the development of T2D. However, T2D develops only if there is dysfunction of beta-cells [4]. In the absence of beta-cell dysfunction individuals can compensate indefinitely for resistance to insulin action with the appropriate hyperinsulinemia. Therefore, many people with remarkable resistance to insulin may never develop T2D [5, 6].

Lifestyle factors clearly underlie MetS incidence, but genetic susceptibility may be important as well [7]. For example, specific ethnic groups are more susceptible to MetS than others [8, 9]. In particular, South Asians are predisposed to develop MetS and subsequently T2D and cardiovascular disease at a younger age [10–12]. They also have a higher prevalence of abdominal obesity, are less sensitive to insulin, and have a lower glucose disposal rate than Europeans [12–14]. In addition, South Asians have lower plasma levels of HDL and adiponectin and higher levels of glucose, insulin, leptin, complement C3, plasminogen activator inhibitor-1, fibrinogen, and tissue plasminogen activator compared to Europeans [15–20]. However, traditional risk factors such as smoking, hypertension, and dyslipidemia do not explain the increased risk for cardiovascular disease in South Asians [21, 22]. Insulin resistance itself has been held responsible for the high rates of T2D and cardiovascular disease in this ethnic group [10, 12].

Oral minimal modeling is a pharmacokinetic/pharmacodynamic algorithm developed to estimate beta-cell function and insulin sensitivity index (ISI) from dynamic data [23]. In the present study, we used this oral minimal model (OMM) to investigate the relationship between MetS traits and beta-cell function in South Asian and European families with prevalent T2D.

## 2. Materials and Methods

### 2.1. Subjects

The recruitment of patients with T2D and their relatives at our university outpatient clinic has been described in detail previously [24]. In brief, 48 South Asians and 54 Europeans that are residing in the Netherlands were initially recruited for the present study and we used an oral glucose tolerance test (OGTT) to group the subjects in T2D or noT2D according to the WHO criteria. In addition, the International Diabetes Federation (IDF) criteria were used to define MetS [25]. Four South Asians had T2D but not MetS and were excluded from the study as the number of subjects was too small for meaningful analyses and there were no European counterparts for comparison. Hence, 98 subjects were included in our analyses (44 South Asians and 54 Europeans) from 25 families (25 patients with T2D but not on insulin therapy and 73 relatives) and they were distributed among 3 groups; no metabolic syndrome/no type 2 diabetes mellitus (noMetS/noT2D), metabolic syndrome/no type 2 diabetes mellitus (MetS/noT2D), and metabolic syndrome/type 2 diabetes mellitus (MetS/T2D).

Written informed consent was obtained from all participants. The Erasmus Medical Ethics Review Board approved the study protocol.

### 2.2. Physical Examination

Body height and weight were measured in light clothing without shoes and were used to estimate body mass index. Waist circumference was measured halfway between the lowest rib and the iliac crest while the maximum circumference of the hips was measured in the standing position; from these measurements the waist-to-hip ratio was calculated. Systolic and diastolic blood pressure were measured in the sitting position with an electronic blood pressure monitor (Datascope Accutorr Plus Inc., Montvale, NJ), after five minutes' rest.

### 2.3. Samples and Measurements

All participants underwent a 210 min OGTT. A 75 g glucose load was administered (*t* = 0), after an overnight fast, and 11 venous blood samples were acquired at prespecified time intervals (−60 min, −15 min, 15 min, 30 min, 45 min, 60 min, 90 min, 120 min, 150 min, 180 min, and 210 min) for the measurement of plasma glucose, insulin, and C-peptide levels. Baseline blood samples were obtained in order to estimate glucose, insulin, C-peptide plasma concentrations, and the lipid profile.

Plasma glucose was estimated using a hexokinase-based method (Gluco-quant; Roche Diagnostics, Mannheim, Germany). Plasma insulin and C-peptide were measured separately by a competitive chemiluminescent immunoassay, supplied by Euro/DPC. The assay was performed on a DPC Immulite 2000 analyzer (Euro/DPC) according to the manufacturer's recommended protocol. Serum total cholesterol, HDL, LDL, and triglycerides were determined with an automatic enzymatic procedure by Roche Diagnostics (Mannheim, Germany).

### 2.4. Oral Minimal Model Calculations

The OMM, which consists of the glucose OMM and the C-peptide OMM, was used to describe changes of plasma glucose, insulin, and C-peptide concentrations during an oral glucose stimulus [26–28]. Glucose, C-peptide, and insulin concentrations were measured at 11 time points before and after intake of 75 g glucose. The glucose OMM estimated ISI with plasma glucose and insulin concentrations measured during the OGTT. In addition the C-peptide OMM indices were calculated during the oral glucose tolerance test and in terms of insulin secretion can be interpreted as follows: (1) the basal (Φ_basal_) that gives a basal nonstimulated measurement of insulin secretion, (2) the dynamic (Φ_dynamic_) that provides the amount of insulin secreted during the dynamic phase (first-phase secretion by beta-cells), (3) the static (Φ_static_) that assesses the release of insulin that occurs after a time delay (second-phase secretion by beta-cells) and represents a beta-cell response according to the prevailing glucose concentration, and (4) the total (Φ_total_) overall secretion which is the sum of the dynamic and static phase release of insulin from beta-cells. The parameters of glucose OMM and C-peptide OMM were multiplied to obtain the disposition indices (DIs), which is a measure for beta-cell function corrected for insulin sensitivity. It can be considered a measure of the functionality of the pancreas in the intact individual: DI_basal_ = Φ_basal_ × ISI, DI_dynamic_ = Φ_dynamic_ × ISI, DI_static_ = Φ_static_ × ISI, and DI_total_ = Φ_total_ × ISI. We performed the analyses with SAAMII software [29].

### 2.5. Statistical Analysis

Continuous variables are expressed as mean ± SEM, unless indicated otherwise. ANOVA test was used to compare the mean of raw data presented in Table 1 and the figures of different subgroups within the two ethnicities. Adjusted analyses were performed with multiple linear regression models in which all covariables were entered synchronously.

A *P* < 0.05 was considered statistically significant. All statistical tests were conducted with SPSS software, version 20 (SPSS Inc., Chicago, IL).

## 3. Results

### 3.1. Baseline Characteristics

32 out of 44 South Asians (72.7%, 95% CI 58.0 to 83.8%) and 23 out of 54 Europeans (42.6%, 95% CI 30.3 to 55.9%) had MetS according to the IDF criteria. In Table 1, the general characteristics of the 3 subgroups are shown according to ethnicity. The systolic blood pressure, fasting, and 2 h plasma glucose levels were increased in South Asians with MetS and T2D compared to the noMetS/noT2D group. South Asians with MetS and T2D were on average 10.4 years younger than Europeans with MetS and T2D. Compared to the analyses of the South Asian subgroups, we observed a larger heterogeneity among the 3 subgroups of the Europeans in clinical and biochemical characteristics with significant differences in age, weight, body mass index, waist, waist-to-hip ratio, blood pressure, and fasting and 2 h plasma glucose levels.

### 3.2. Insulin Sensitivity

**Figure 1:**
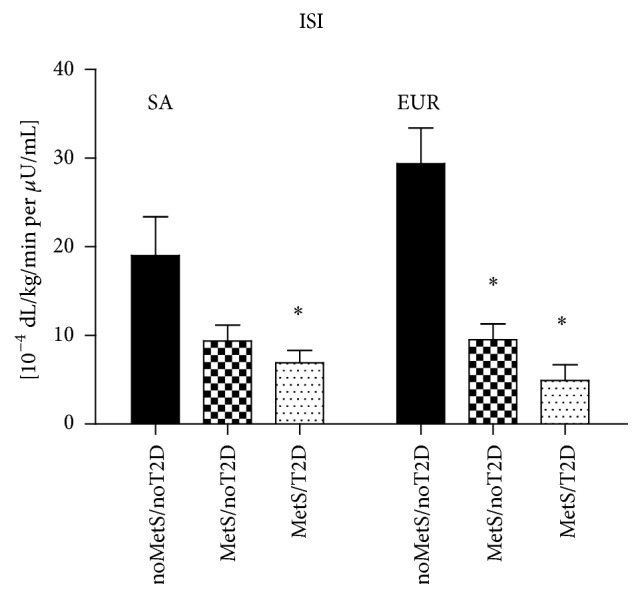
Insulin sensitivity index (ISI) assessed by the OMM in South Asian (SA) and European (EUR) subgroups. *∗* indicates a statistically significant difference between noMetS/noT2D and other subgroups.

The ISIs of the three subgroups according to ethnicity are shown in Figure 1. Subjects without MetS had higher ISI than those with MetS. On average and adjusted for sex and age, the Europeans had 7.18 × 10^−4^ dL/kg/min per *μ*U/mL (95% CI 0.58 to 13.78, *P* = 0.033) higher ISI than the South Asians. This was fully explained by the differences between the two subgroups without MetS (after excluding this subgroup to the analysis the effect of ethnicity disappeared, *P* = 0.367).

### 3.3. Beta-Cell Responsivity Indices

The fitting of both C-peptide and glucose OMM was satisfactory as the average weighted residuals did not deviate systematically from the zero value (Supplementary Figures  1 and 2 in Supplementary Material available online at http://dx.doi.org/10.1155/2016/9286303).

**Figure 2:**
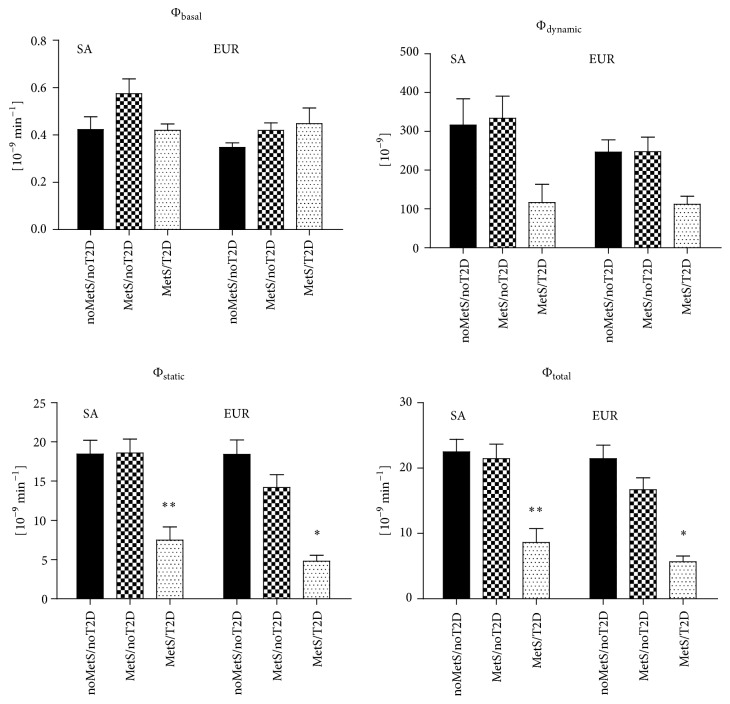
*β*-cell insulin responsivity indices in South Asian (SA) and European (EUR) subgroups. *∗* indicates a statistically significant difference in comparison with the noMetS/noT2D subgroup; *∗∗* indicates a statistically significant difference between the MetS/noT2D and MetS/T2D subgroup.

Figure 2 shows the beta-cell responsivity indices estimated by the C-peptide OMM during the OGTT. Adjusted for sex and age, ethnicity was significantly related to the basal responsivity index, *β* = 0.086 × 10^−9^/min, 95% CI 0.17 to 0.155, and *P* = 0.016, but not to the other responsivity indices, *P* > 0.7. Within the South Asian group adjusted for sex and age, the MetS/noT2D group had 0.164 × 10^−9^/min (95% CI 0.045 to 0.282, *P* = 0.008) higher basal responsivity index compared to the other subgroups. The basal responsivity index was not significantly different between the European subgroups. The dynamic, the static, and the total responsivity indices showed all the same trend of a lower beta-cell response (in effect less insulin secretion) in the MetS/T2D groups in both ethnicities: −191.9 × 10^−9^ (95% CI −282.2 to −101.6, *P* = 0.001), −12.1 × 10^−9^/min (95% CI −16.1 to −8.0, *P* = 0.001), and −14.3 × 10^−9^/min (95% CI −18.9 to −9.6, *P* = 0.001), respectively. The South Asian noMetS/noT2D and MetS/noT2D had similar values in the dynamic, the static, and the total responsivity indices, whereas in the Europeans there was a clear trend to gradually lower values over these groups in the direction of the MetS/T2D subgroup: *P*_for  trend_ = 0.005, 0.001, and 0.001, respectively.

### 3.4. Disposition Indices

**Figure 3:**
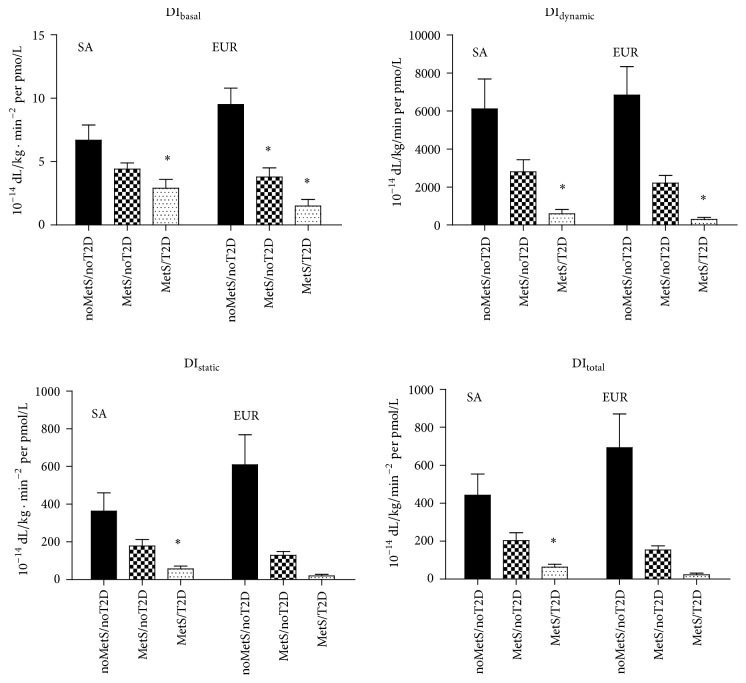
Disposition indices in South Asian (SA) and European (EUR) subgroups. *∗* indicates a statistically significant difference in comparison with the noMetS/noT2D subgroup.

The DIs according to subgroup and ethnicity are shown in Figure 3. Adjusted for sex and age the four DIs did not differ significantly between the ethnicities (*P* > 0.11). Both ethnicities showed the same gradual course of the different DI from high to intermediate and low over the noMetS/noT2D, MetS/noT2D, and MetS/T2D subgroups, respectively (*P*_for  trend_ < 0.002). There were no clear differences between the ethnicities, although the European noMetS/noT2D subgroup had relatively high DI compared to the same South Asian subgroup.

### 3.5. MetS Traits and OMM Indices

The relationships between the MetS traits and OMM indices adjusted for sex and age according to ethnicity are shown in Table 2. The MetS traits were not used in a large “holistic” multiple linear regression model since they are correlated. Therefore, each trait was analyzed separately in a multiple linear regression model adjusted for sex and age. Waist-to-hip-ratio was strongly associated with ISI in both ethnicities, but it may have a more detrimental effect of the glucose disposition in Europeans than in the South Asians. The dyslipidemia characterized by high triglycerides and low HDL was related to ISI and the DIs solely in the South Asians, whereas blood pressure, both systolic and diastolic, was solely related to ISI and the DIs in the Europeans. As expected, fasting glucose was associated with ISI and DIs as these indices are calculated with glucose values.

## 4. Discussion

In the present study, we found a clear trend for increasing insulin resistance from noMetS/noT2D to MetS/noT2D and to MetS/T2D. We also found that South Asians without MetS were more resistant to insulin than a corresponding group of Europeans. In the European families, the dynamic, static, and total beta-cell responsivity indices followed the trend of ISI in the same direction over the three subgroups towards decreasing insulin secretion by the beta-cells in the T2D group. The South Asian noMetS/noT2D and MetS/noT2D subgroups both had high beta-cell responsivity indices and the MetS/T2D subgroup had low values. The DIs are the product of ISI and beta-cell responsivity indices and independent of ethnicity followed a decreasing trend over the subgroups in the direction of T2D. Notably, a number of separate MetS traits had different associations in the two ethnicities: dyslipidemia had a strong relationship with ISI and DI in the South Asians, whereas blood pressure was associated with ISI and DI in the Europeans.

Over the last years several studies have examined the effect of lifestyle, dietary habits, and genetic polymorphisms on the development of MetS in different ethnicities, in order to identify high-risk populations such as the South Asians, who appear to have an increased predisposition for T2D [30–32]. Most of these reports focused on epidemiological analyses and evaluations of the impact of biochemical and physical characteristics (e.g., total abdominal fat and intra-abdominal adipose tissue) on the development of MetS. In a number of studies, ISI and beta-cell function were assessed in different ethnicities using fasting blood glucose and plasma insulin concentrations (for instance, homeostatic model assessment (HOMA)) [33–35]. These methods do not assess the dynamic response of beta-cells to glucose stimuli [36, 37]. Our study is the first to employ OMM in order to examine the relationships between MetS traits and ISI and beta-cell response. The estimations of ISI and insulin secretion by OMM are reasonably well correlated with those assessed by the hyperinsulinemic euglycemic clamp method [38].

We demonstrated for the first time, to our knowledge, that in South Asians and Europeans both ISI and beta-cell function are lower in subjects with MetS and in subjects with MetS and T2D compared to noMetS/noT2D subjects. In addition, we confirmed an increased insulin resistance in noMetS/noT2D South Asians compared to noMetS/noT2D Europeans [39, 40]. Moreover, in South Asians with noMetS/noT2D the disposition indices were lower comparing to the disposition indices in Europeans and not statistically different from the disposition indices in South Asians with MetS/noT2D. These findings indicate that South Asians are a vulnerable population and have an increased risk to develop chronic diseases such as MetS and T2D, in which insulin resistance and beta-cell function are key pathogenetic factors.

Our study showed that OMM could be used to evaluate glucose homeostasis in different populations and to examine associations between insulin resistance, abnormal beta-cell function, and MetS traits. Waist-to-hip ratio reflects the association between visceral obesity and the causal path from insulin resistance to T2D [41–43]. Hypertriglyceridemia and increased enzymes involved in triglyceride transport and metabolism may have a toxic effect on beta-cells [44], but hypertriglyceridemia can be a consequence of insulin resistance and T2D as well. Remarkably, triglycerides and HDL were associated with ISI and DIs in South Asians. Unfortunately, we cannot infer from our data whether dyslipidemia was the cause or the consequence of insulin resistance and reduced DIs. In the European families, insulin resistance and reduced DI were associated with raised blood pressure. This suggests that disorders of carbohydrate metabolism were complicated by higher levels of blood pressure. A possible explanation is an impaired insulin signaling through PI3K signaling pathway and the increased vascular inflammation noted in insulin resistance which can decrease nitric oxide (NO) production and increase Endothelin-1 (ET-1) secretion leading to raised blood pressure [45, 46]. The differences in the associations between metabolic syndrome traits, beta-cell function, and insulin sensitivity in different ethnicities have not been fully explained yet. A possible etiology is the different genetic profile of Europeans and South Asians; however, further research is required towards this direction in order to understand the metabolic pathways and the effect of the genome on these associations.

In the present analysis, we investigated associations between metabolic syndrome traits and insulin sensitivity and beta-cell function after adjusting for age and sex. Of note the results did not change significantly when we adjusted also for BMI. The only differences that we noticed were in Europeans where there was no longer an association between ISI, waist-to-hip ratio, and diastolic blood pressure as well as a correlation between fasting glucose and ISI and DI_static_ (Supplementary file Table  1).

### 4.1. Strengths and Limitations

We have recruited subjects through families with prevalent T2D. Family studies avoid a number of serious selection biases, but the findings cannot easily be generalized to the general population. Moreover, we prefer to analyze family data using a family matrix, for instance, SOLAR, but unfortunately there were too many small cells. Adjustment for family ties in regression analyses suffered from the same problem; therefore, we restricted the adjustment of all primary analyses to age, which correlates with generation in kindreds. We did not analyze genetic variation, and transmission was not a topic of our research. Moreover, separate analyses of small and large families did not change the results (data not shown).

We chose the comparison between the MetS/T2D status groups and not between the two ethnic groups because South Asians and Europeans differ in many aspects and therefore we tried to avoid bias due to the ethnicity differences.

We have used the IDF criteria for MetS, because they take ethnic specific cut-off points for central obesity, in effect waist circumference, into account. South Asians residing in the Western world differ in lifestyle and phenotype from the South Asians living in South Asia, and therefore we tested the necessity of such ethnicity-specific cut-off points: if the European cut-off points were used for all subjects, only three male South Asians would have not been classified as MetS. Still a number of arguments could be made to include analyses using a single cut-off point, but the effects on our results were negligible (data not shown) and therefore we decided to follow the IDF recommendations.

A limitation of the study was the relatively small sample size and for this reason these findings cannot be generalized to the broader community. Furthermore, we excluded the noMetS/T2D subgroup, because meaningful analysis was not possible, as there were only 4 South Asians and no Europeans counterparts.

## 5. Conclusions

In our study, we observed low ISIs in the MetS/T2D group relative to the noMetS/noT2D group in both ethnicities, a finding that indicates an association between insulin resistance and a pathologic metabolic state (T2D). The pathogenic mechanisms that lead to insulin resistance appear to differ among ethnicities, as ISI and DIs were associated with different metabolic traits in each ethnicity. The simultaneous assessment of insulin sensitivity and beta-cell function allowed us to study metabolic profiles that promote T2D and glucose intolerance. We used this primarily to explore the underlying mechanisms of disturbances in glucose metabolism, but also to explore the potential of designing ethnicity-specific risk models for early identification of subjects that are at risk for developing diabetes mellitus. In this study, South Asians, in contrast to Europeans, appear to be predisposed to insulin resistance and exhibit impaired beta-cell function at an earlier age. Dyslipidemia related to insulin resistance and pancreatic dysfunction may underlie or enhance this susceptibility of South Asians.

## Supplementary Material

The supplementary file includes 2 figures and 1 table. Figure 1 and 2 illustrate the weighted residuals for C-peptide and Glucose Oral Minimal Model, respectively. The table shows the relationship between metabolic syndrome traits and insulin sensitivity index (ISI), dynamic disposition index (DIdynamic) and static disposition index (DIstatic) adjusted for sex, age and BMI according to ethnicity.

## Figures and Tables

**Table 1:** General characteristics of the study subjects.

	South Asians (*n* = 44)			Europeans (*n* = 54)		
	noMetS/noT2D	MetS/noT2D	MetS/T2D	noMetS/noT2D	MetS/noT2D	MetS/T2D
*N*	12	14	18	31	12	11
Male/female	6/6	9/5	10/8	9/22	5/7	6/5
Age (yrs)	33.9 ± 8.0	48.2 ± 10.1^‡^	51.4 ± 10.4^‡^	39.6 ± 10.2	40.3 ± 10.2	61.8 ± 8.7^**∗**†^
Height (m)	1.67 ± 0.12	1.68 ± 0.12	1.63 ± 0.08	1.75 ± 0.10	1.78 ± 0.08	1.77 ± 0.07
Weight (kg)	74.1 ± 16.3	78.8 ± 9.6	76.3 ± 12.4	77.8 ± 12.2	103.5 ± 28.7^**∗**^	98.3 ± 11.8^**∗**^
Body mass index (kg/m^2^)	26.3 ± 3.3	28 ± 2.7	28.7 ± 4.1	25.4 ± 3.1	32.6 ± 8.2^**∗**^	31.4 ± 4.3^**∗**^
Waist (cm)	93 ± 12	98 ± 9	97 ± 7	88 ± 12	110 ± 19^**∗**^	110 ± 13^**∗**^
Hip (cm)	105 ± 7	104 ± 4	103 ± 8	106 ± 8	120 ± 16^**∗**^	116 ± 8
Waist-to-hip ratio	0.89 ± 0.08	0.94 ± 0.07	0.95 ± 0.08	0.83 ± 0.09	0.92 ± 0.08^**∗**^	0.94 ± 0.07^**∗**^
Systolic blood pressure (mmHg)	114 ± 11	130 ± 16	136 ± 15^‡^	120 ± 12	137 ± 14^**∗**^	137 ± 14^**∗**^
Diastolic blood pressure (mmHg)	76 ± 9	83 ± 12	82 ± 9	74 ± 8	84 ± 9^**∗**^	84 ± 11^**∗**^
Total cholesterol (mmol/L)	4.7 ± 0.7	5 ± 0.9	4.6 ± 1.2	4.8 ± 1	4.9 ± 0.9	4.5 ± 1.1
Triglycerides (mmol/L)	0.97 ± 0.37	1.43 ± 0.65	1.55 ± 0.65	0.97 ± 0.36	1.39 ± 0.77	1.38 ± 0.55
HDL (mmol/L)	1.19 ± 0.37	1.01 ± 0.21	1.05 ± 0.22	1.47 ± 0.40	1.11 ± 0.19^**∗**^	1.17 ± 0.26
LDL (mmol/L)	3.02 ± 0.63	3.30 ± 0.76	2.80 ± 1.08	2.77 ± 0.93	3.01 ± 0.80	2.55 ± 0.90
Ratio HDL-cholesterol	4.2 ± 1.3	5.2 ± 1.4	4.4 ± 1.2	3.5 ± 1.1	4.5 ± 1.2^**∗**^	3.9 ± 0.9
Fasting plasma glucose (mmol/L)	5.3 ± 0.3	5.8 ± 0.5	7.4 ± 1.5^§‡^	5.3 ± 0.5	5.7 ± 0.5	8.1 ± 1.1^**∗**†^
OGTT (mmol/L)	5.5 ± 1.7	6.8 ± 1.2	13.8 ± 5.2^§‡^	5.7 ± 1.5	6.7 ± 1.8	13.9 ± 2.8^**∗**†^
Insulin (pmol/L)	53.5 ± 46.8	73.8 ± 49.2	58.3 ± 38.4	35.8 ± 29	73.7 ± 35.6	78.5 ± 72.2

HDL: high-density lipoprotein; LDL: low-density lipoprotein; OGTT: oral glucose tolerance test (2 hours); MetS: metabolic syndrome; T2D: type 2 diabetes mellitus.

**∗** indicates statistically significant differences between the noMetS/noT2D and the other 2 subgroups within Europeans; † indicates statistically significant differences between the MetS/noT2D and the Met/T2D subgroup within Europeans; ‡ indicates statistically significant differences between noMetS/noT2D and the other 2 subgroups within South Asians; § indicates statistically significant differences between MetS/noT2D and the Met/T2D subgroup within South Asians.

**Table 2:** Relationship between metabolic syndrome traits and insulin sensitivity index (ISI), dynamic disposition index (DI_dynamic_), and static disposition index (DI_static_) adjusted for sex and age according to ethnicity.

Metabolic syndrome trait	ISI	95% CI	*P*	DI_dynamic_	95% CI	*P*	DI_static_	95% CI	*P*
	×10^−4^ dL/kg/min per *μ*U/mL			×10^−10^ dL/kg/min per *μ*U/mL			×10^−10^ dL/kg/min per *μ*U/mL		
*South Asians*									
Waist-to-hip ratio	−61	−109 to −13	0.015	−9.4	−27.0 to 8.1	0.284	−0.7	−1.8 to 0.3	0.181
Triglycerides (mmol/L)	−7.1	−11.9 to −2.3	0.005	−2.0	−3.7 to −0.3	0.022	−0.1	−0.2 to −0.04	0.007
HDL (mmol/L)	25.7	15.8 to 35.7	0.001	5.9	1.9 to 9.9	0.005	0.5	0.2 to 0.7	0.001
Systolic blood pressure (mmHg)	−0.2	−0.4 to −0.03	0.028	0.06	−0.1 to 0.02	0.129	−0.005	−0.009 to −0.0006	0.026
Diastolic blood pressure (mmHg)	−0.2	−0.5 to 0.1	0.169	−0.04	−0.1 to 0.07	0.487	−0.003	−0.01 to 0.003	0.281
Fasting plasma glucose (mmol/L)	−3.0	−5.3 to −0.6	0.014	−1.2	−2.0 to −0.4	0.003	−0.07	−0.1 to −0.003	0.002
*Europeans*									
Waist-to-hip ratio	−95	−159 to −31	0.005	−25.6	−49.6 to −1.7	0.036	−22.9	−4.8 to −0.2	0.075
Triglycerides (mmol/L)	−7.9	−18 to 2.4	0.131	−0.3	−4.0 to 3.5	0.888	−0.1	−0.5 to 0.3	0.518
HDL (mmol/L)	10.6	−5.9 to 27.1	0.204	1.3	−4.7 to 7.3	0.668	0.3	−0.3 to 1.0	0.266
Systolic blood pressure (mmHg)	−0.6	−0.9 to −0.3	0.001	−0.1	−0.3 to −0.02	0.026	−0.02	−0.03 to −0.006	0.005
Diastolic blood pressure (mmHg)	−0.8	−1.3 to −0.3	0.004	−0.1	−0.3 to 0.05	0.144	−0.02	−0.04 to 0.004	0.111
Fasting plasma glucose (mmol/L)	−5.2	−10.3 to −0.09	0.046	−2.3	−4.1 to −0.5	0.01	−0.2	−0.4 to 0.005	0.056

HDL: high-density lipoprotein; ISI: insulin sensitivity index; DI_dynamic_: dynamic disposition index; DI_static_: static disposition index.
