# Supplementary material for: The Relationship of Metabolic Syndrome Traits with Beta-Cell Function and Insulin Sensitivity by Oral Minimal Model Assessment in South Asian and European Families Residing in the Netherlands
Source: J Diabetes Res. 2016 Aug 11;2016:9286303. doi: 10.1155/2016/9286303 (PMC4997024; doi:10.1155/2016/9286303)
Supplement: Supplementary file 1 — The supplementary file includes 2 figures and 1 table. Figure 1 and 2 illustrate the weighted residuals for C-peptide and Glucose Oral Minimal Model, respectively. The table shows the relationship between metabolic syndrome traits and insulin sensitivity index (ISI), dynamic disposition index (DIdynamic) and static disposition index (DIstatic) adjusted for sex, age and BMI according to ethnicity. [file 9286303.f1.docx]

**Supplementary Figure 1**. Average weighted residuals for the C-peptide oral minimal model

**Supplementary Figure 2.** Average weighted residuals for the glucose oral minimal model

**Supplementary Table 1.** Relationship between metabolic syndrome traits and insulin sensitivity index (ISI), dynamic disposition index (DI_dynamic_) and static disposition index (DI_static_) adjusted for sex, age and BMI according to ethnicity

| **Metabolic syndrome trait** | | **ISI** | | **95% CI** | | **P** | | **DI_dynamic_** | | **95% CI** | | **P** | | **DI_static_** | | **95% CI** | | **P** | | |  |
| --- | --- | --- | --- | --- | --- | --- | --- | --- | --- | --- | --- | --- | --- | --- | --- | --- | --- | --- | --- | --- | --- |
|  | | x10^-4^ dl/kg/min per μU/ml | | | | | | x10^-10^ dl/kg/min per μU/ml | | | | | | x10^-10^ dl/kg/min per μU/ml | | | | | | |  |
| **South Asians** | | |  | |  | |  | |  | |  | |  | |  | |  | |  | |  |
|  | Waist-to-hip ratio | | -60.75 | | -109 to -12.1 | | 0.016 | | -9.03 | | -26.56 to 8.5 | | 0.303 | | -0.694 | | -1.74 to 0.36 | | | 0.189 | |
|  | Triglycerides (mmol/L) | | -6.72 | | -11.53 to -1.91 | | 0.007 | | -1.84 | | -3.52 to -0.16 | | 0.032 | | -0.130 | | -0.23 to -0.031 | | | 0.012 | |
|  | HDL (mmol/L) | | 24.6 | | 14.12 to 35.08 | | 0.000 | | 5.2 | | 1.17 to 9.38 | | 0.013 | | 0.435 | | 0.21 to 0.664 | | | 0.000 | |
|  | Systolic blood pressure (mmHg) | | -0.21 | | -0.44 to 0.023 | | 0.077 | | -0.04 | | -0.12 to 0.04 | | 0.322 | | -0.004 | | -0.009 to 0.000 | | | 0.074 | |
|  | Diastolic blood pressure (mmHg) | | -0.22 | | -0.53 to 0.087 | | 0.154 | | -0.04 | | -0.15 to 0.07 | | 0.457 | | -0.004 | | -0.01 to 0.003 | | | 0.259 | |
|  | Fasting plasma glucose (mmol/L) | | -2.73 | | -5.06 to -0.4 | | 0.023 | | -1.11 | | -1.87 to -0.35 | | 0.005 | | -0.069 | | -0.11 to -0.023 | | | 0.004 | |
|  |  | |  | |  | |  | |  | |  | |  | |  | |  | | |  | |
| **Caucasians** | | |  | |  | |  | |  | |  | |  | |  | |  | | |  | |
|  | Waist-to-hip ratio | | -47.1 | | -117.14 to 22.94 | | 0.183 | | -16 | | -43.48 to 11.45 | | 0.248 | | -1.162 | | -4.054 to 1.73 | | | 0.423 | |
|  | Triglycerides (mmol/L) | | -6.9 | | -15.98 to 2.2 | | 0.134 | | -0.039 | | -3.675 to 3.6 | | 0.983 | | -0.104 | | -0.48 to 0.275 | | | 0.583 | |
|  | HDL (mmol/L) | | 0.87 | | -14.94 to 16.68 | | 0.912 | | -1.072 | | -7.24 to 5.098 | | 0.728 | | 0.130 | | -0.514 to 0.774 | | | 0.687 | |
|  | Systolic blood pressure (mmHg) | | -0.43 | | -0.76 to -0.100 | | 0.012 | | -0.099 | | -0.23 to 0.036 | | 0.146 | | -0.015 | | -0.029 to -0.001 | | | 0.036 | |
|  | Diastolic blood pressure (mmHg) | | -0.4 | | -0.93 to 0.137 | | 0.141 | | -0.05 | | -0.26 to 0.163 | | 0.637 | | -0.007 | | -0.029 to 0.015 | | | 0.529 | |
|  | Fasting plasma glucose (mmol/L) | | -3.27 | | -7.98.3 to 1.44 | | 0.169 | | -1.95 | | -3.74 to -0.158 | | 0.034 | | -0.144 | | -0.336 to 0.047 | | | 0.137 | |
